# Twenty-nine newly sequenced genomes and a comprehensive genome dataset for the insect endosymbiont *Buchnera*

**DOI:** 10.1038/s41597-024-03537-0

**Published:** 2024-06-22

**Authors:** Congcong Lu, Tianmin Zou, Qian Liu, Xiaolei Huang

**Affiliations:** State Key Laboratory of Ecological Pest Control for Fujian and Taiwan Crops, College of Plant Protection, Fujian Agriculture and Forestry University, Fuzhou, 350002 China

**Keywords:** Chromosomes, Molecular evolution

## Abstract

Most phloem-feeding insects face nutritional deficiency and rely on their intracellular symbionts to provide nutrients, and most of endosymbiont genomes have undergone reduction. However, the study of genome reduction processes of endosymbionts has been constrained by the limited availability of genome data from different insect lineages. The obligate relationship between aphids and *Buchnera aphidicola* (hereafter *Buchnera*) makes them a classic model for studying insect-endosymbiont interaction. Here, we report 29 newly sequenced *Buchnera* genomes from 11 aphid subfamilies, and a comprehensive dataset based on 90 *Buchnera* genomes from 14 aphid subfamilies. The dataset shows a significant genomic difference of *Buchnera* among different aphid lineages. The dataset exhibits a more balanced distribution of *Buchnera* (from 14 aphid subfamilies) genome sizes, ranging from 400 kb to 600 kb, which can illustrate the genome reduction process of *Buchnera*. The new genome data provide valuable insights into the microevolutionary processes leading to genomic reduction of insect endosymbionts.

## Background & Summary

Insects are known to be associated with multiple symbionts for acquiring unique and beneficial functions^1^ during the 480 million years of evolutionary history^2,3^. Symbionts can help the host insect in better adapting to complex and dynamic ecological environments, influencing mating, reproduction, metabolism, and immunity of hosts^2^. These symbionts participate in many life activities of their host insects, for example, helping insects in resisting the invasion of pathogenic microorganisms and parasites, evading predators, developing resistance to insecticides, and synthesizing essential nutrients required by insects^4^. Furthermore, the composition and metabolic activities of symbionts are influenced by the selection and regulation of the host insects. Symbionts are considered as a unique “multifunctional organ” of insects and an indispensable component of insects. The study of insect symbionts has significant implications in biocontrol, interruption of vector-borne diseases, and prevention and control of insect pests^5^.

The phenomenon of genome reduction has been observed consistently across various obligate symbionts in insects, particularly within the suborder Sternorrhyncha. This symbiont is strictly dependent on the host, transmitted exclusively maternally, and has co-diversified with hosts for a significant duration, undergoing early genome loss yet often maintaining stability over time^6,7^. Examples include *Carsonella ruddii* in psyllids (158–166 kb)^8^, *Portiera aleyrodidarum* in whiteflies (281–358 kb)^9^, *Tremblaya princeps* (139–171 kb) and *Moranella endobia* (538 kb) in mealybugs^10–12^, as well as *Buchnera aphidicola* in aphids (with genome sizes ranging from 419 to 656 kb)^13^. The aphid-*Buchnera* system is a classic model to investigate insect-endosymbiont interaction. Aphids, feeding on phloem sap as their dietary source, face nutritional limitations due to the rich in simple sugars but unbalanced mixture of amino acids in sap^14,15^. Therefore, nearly all aphids rely on their specialized intracellular symbiotic bacteria, *Buchnera aphidicola* (Gammaproteobacteria), to provide them with essential amino acids, vitamins, and other important nutrients^16^. *Buchnera* is exclusively found within specialized bacteriocytes, which are symmetrical arrangements in the abdominal hemocoel of aphids^17,18^. *Buchnera* has been identified only in Aphididae species, and cannot survive independently outside the host aphids^19^. Therefore, expanding the dataset of *Buchnera* genomes is crucial for advancing our understanding of the evolutionary dynamics within these endosymbionts. The additional genomes will enable comprehensive analyses of genetic variation, adaptation, and co-evolutionary patterns, shedding light on fundamental aspects of endosymbionts and host-endosymbionts interactions.

The characteristics of clonality and maternal vertical transmission contribute to a faster fixation rate of slightly deleterious mutations in *Buchnera* than free-living relatives, which is reflected in the accelerated evolution of protein coding genes^20^, gene inactivation and loss^21,22^, leading to a significant reduction in genome size^23,24^. While the *Buchnera* have undergone genome reduction, most of them still retain the necessary genes related to the biosynthesis of essential amino acids required by aphids^22,25,26^. The genome size of *Buchnera* in the Lachninae is only from 422 kb to 458 kb, while in the Macrosiphini of Aphidinae ranges from 614 kb to 671 kb, reflecting the adaptive evolution between aphids and *Buchnera* at the genomic level^13^. Chong *et al*.^27^ analyzed 39 *Buchnera* genomes from 6 aphid subfamilies and found that the most recent common ancestor of *Buchnera* had at least 616 protein coding genes, which then experienced non-random gene loss in different lineages^27^.

With the continuous advancement of high-throughput sequencing technologies, the number of obligate symbiont genomes has increased. Currently, 77 *Buchnera* strains from 61 aphid species belonging to 10 subfamilies have been deposited in the GenBank database. However, the majority of these data are concentrated in Aphidinae, with 49 *Buchnera* strains from 33 species, accounting for approximately 63.6%. And the sizes of the published *Buchnera* genomes are mainly around 400 kb or 600 kb, which do not well represent the process of genome reduction. To thoroughly explore the diversity of *Buchnera* genomes across different aphid species, we sequenced 29 new genomes representing 19 aphid species of 11 subfamilies. This addition includes *Buchnera* genomes from four additional aphid subfamilies (Greenideinae, Mindarinae, Neophyllaphidinae and Taiwanaphidinae). To delve deeper into the reduction patterns of *Buchnera* genomes, a more comprehensive and reliable dataset is needed. Therefore, based on a quality control process, we constructed a robust dataset comprising 90 *Buchnera* genomes by combining the 29 newly sequenced genomes from 11 subfamilies and 61 selected genomes from the GenBank. The dataset we utilized demonstrates a more even distribution of *Buchnera* genome sizes, encompassing sizes such as 400 kb, 500 kb, and 600 kb. This diversity illustrates the ongoing process of genome reduction in *Buchnera*. In contrast, previous datasets predominantly featured *Buchnera* genome sizes concentrated around 400 kb and 600 kb. Additionally, our dataset includes 47 aphid species from a wider range of families, with primary representation from Aphidinae (600 kb) and Lachninae (400 kb). The extensive coverage of genome data can be effectively employed to validate the genome evolution of *Buchnera* within a phylogenetic framework, and contribute to understanding of the microevolutionary processes that shape genome reduction in insect obligate endosymbionts.

## Methods

### Sampling and DNA sequencing

Twenty-nine aphid samples from 11 subfamilies (Aphidinae, Calaphidinae, Chaitophorinae, Drepanosiphinae, Eriosomatinae, Greenideinae, Hormaphidinae, Lachninae, Mindarinae, Neophyllaphidinae, Phyllaphidinae, Taiwanaphidinae and Thelaxinae) were collected from May 2015 to June 2019 in various regions of China, including Fujian, Yunnan, Guangdong, Beijing, Zhejiang, Jiangxi, and Guangxi. All collected aphid specimens were identified to species level mainly based on morphological characters by experienced aphid taxonomists. Some samples from the same aphids originated from different geographical locations, spanning a considerable geographic range, evenly from south to north (Table S1). Each sample comprised of multiple individuals collected from the same aphid colony on a single host plant. Detailed sampling information is listed in the Supplemental Table S1. The specimens were kept in 95% ethanol and store at −20 °C after collection. Due to the different body size of aphid species, five to twenty apterous adult females of each sample were used for DNA extraction. After washing three times in ultrapure water, the genomic DNA extraction from the aforementioned pooled samples of each aphid species were performed using the DNeasy Blood and Tissue kit (QIAGEN), following the manufacturer’s manual.

All DNA samples were sent to Biomarker Technologies Co., Ltd. (China, Beijing) for metagenomic next-generation sequencing. The metagenomic library construction process involved fragmentation and purification of genomic DNA, followed by end repair and A-tailing. Subsequently, adapters were ligated to the fragments, and the resulting products were purified. PCR amplification was performed, and the resulting products were purified again. Finally, the library quality was assessed prior to downstream analysis. Following library construction, the library underwent Illumina (Illumina Corp., San Diego CA, USA) 2 × 150 paired-end sequencing using Illumina NovaSeq 6000 platform (Illumina Corp., San Diego CA, USA) with the NovaSeq 6000 S4 Reagent Kit (Illumina Corp., San Diego CA, USA). To ensure the quality of bioinformatics, raw reads were filtered to obtain clean reads. Trimmomatic^28^ software (parameters: LEADING: 3, TRAILING: 3, SLIDINGWINDOW: 50:20, MINLEN: 100) was used to filter raw tags and obtain high-quality sequencing data (clean tags). Finally, a minimum of 10 GB of sequence data (clean reads) was generated for each sample.

### Genome assembly and annotation

Clean reads were utilized to assemble the genome of *Buchnera*. The assembly process began with the use of MEGAHIT v1.1.1^29^, which generated numerous long contigs in a final assembly fasta document. Subsequently, 16S rRNA sequences of *Buchnera* from the same species of 29 aphids available on NCBI were downloaded. These 16S rRNA sequences served as query sequences to extract high-coverage *Buchnera* chromosome genomes from the final assembly sequences using BLASTN v2.5.0^30^. The extracted sequences were then de novo assembled using Geneious v7.1.9^31^. In cases where the genome was not circular, the extracted sequence served as a seed sequence for subsequent assembly in NOVOPlasty v3.5^32^ (parameters: Insert size = 250, Read length = 150, Genome range = 40000–70000, K-mer = 33, 30, 27). Most of the *Buchnera* genomes were circularized through this process. For cases where circularization was not achieved, sequences from NOVOPlasty and MEGAHIT were combined and assembled together in Geneious. Finally, all *Buchnera* genomes were successfully circularized. The circular draft genome sequences were corrected by Pilon^33^ for bases correcting and mis-assemble fixing, based on the paired end data.

We additionally assembled the mitochondrial genomes (mitogenomes) of host aphids using the aforementioned methods. For this purpose, we utilized the *cox1* sequences and complete mitogenome sequences of closely related species as seed sequences.The mitogenomes of *Kurisakia onigurumii* Mt23 & Mt24, *Neophyllaphis podocarpi* Mt25, and *Neophyllaphis varicolor* Mt26 were not circular and were represented by a long contig, respectively. However, we successfully assembled the mitogenomes of the remaining 25 host aphids into circular genomes.

Another 61 *Buchnera* genomes from the Genbank were selected and incorporated with our newly sequenced genomes into a comprehensive genome dataset of 90 *Buchnera* genomes. Among the 77 genomes accessible on NCBI (https://www.ncbi.nlm.nih.gov/datasets/genome/?taxon=9, 2022), we specifically chose 61 *Buchnera* genomes originating from diverse aphid hosts. In particular, only one *Buchnera* strain per aphid species was retained, typically favoring strains that had undergone detailed genome analysis and possessed the relative large genomes. Additionally, *Buchnera* genome sequences with high levels of degenerate bases (more than 5%) were not considered, despite species of this kind having only one *Buchnera* genome. For example, *Hormaphis cornu* isolate DLS_fromHcor80 (accession number: CP051840.1) has a total genome length of 643,231 bp, but it contains 60,999 degenerate bases “N”, accounting for 9.48% of the sequence. Such instances could potentially mislead future analyses related to genome structure and features. The names of all *Buchnera* strains are presented by the species name of their corresponding host aphids (Table 1). All complete circular genome were annotated through both the RAST Server v2.0^34^ and Prokka v1.13.3^35^. All genes that differed in length or location were manually curated. The gene names were standardized based on the Prokka annotation.

**Table 1:** Sources and genome characteristics (base composition, gene size, and gene number) of 90 *Buchnera* strains used in dataset construction.

*Buchnera*	Host aphids	Source	Base composition								Genome components size (bp)						Gene count			
			A (%)	T (%)	C (%)	G (%)	A + T (%)	G + C (%)	AT skew	GC skew	Genome	CDS	tRNA	rRNA	tmRNA	Noncoding	CDS	tRNA	rRNA	tmRNA
1	*Muscaphis stroyani*	CP034861.1	37.3	36.9	12.8	12.9	74.2	25.7	0.006	0.004	619238	5E + 05	2461	4557	368	77039	565	32	3	1
2	*Melanaphis sacchari*	CP029161.1	37.2	37.5	12.7	12.6	74.7	25.3	−0.005	−0.004	626137	6E + 05	2554	4562	375	65065	581	33	3	1
3	*Schizaphis graminum*	CP029205.1	37.2	37.5	12.8	12.5	74.7	25.3	−0.004	−0.01	641689	6E + 05	2472	4562	373	69646	599	32	3	1
4	*Rhopalosiphum maidis*	CP032759.1	37.2	37.5	12.8	12.5	74.7	25.3	−0.004	−0.01	642929	6E + 05	2467	4553	376	66277	602	32	3	1
5	*Rhopalosiphum padi*	CP034858.1	37.5	37.2	12.5	12.7	74.7	25.2	0.004	0.008	643941	6E + 05	2475	4558	375	65087	594	32	3	1
6	*Aphis nerii*	CP034885.1	38.1	37.8	12.1	12.1	75.9	24.2	0.004	0.001	631491	6E + 05	2465	4553	369	61991	589	32	3	1
7	*Aphis helianthi*	CP034894.1	38.1	37.8	12	12.1	75.9	24.1	0.004	0.001	634211	6E + 05	2471	4548	369	59982	591	32	3	1
8	*Aphis aurantii* 13	*CP135018	37.6	37.3	12.5	12.6	74.9	25.1	0.004	0.001	629080	6E + 05	2465	4552	369	64084	578	32	3	1
9	*Aphis aurantii* 14	*CP135021	37.6	37.3	12.5	12.6	74.9	25.1	0.004	0.001	628870	6E + 05	2463	4552	369	62784	575	32	3	1
10	*Aphis craccivora*	CP043999.1	37.9	37.6	12.2	12.2	75.5	24.4	0.004	0.003	632731	6E + 05	2468	4546	369	66565	579	32	3	1
11	*Aphis fabae*	CP042427.1	38	37.7	12.1	12.1	75.7	24.2	0.004	0.004	634931	6E + 05	2468	4549	371	61212	589	32	3	1
12	*Aphis urticata*	CP048744.1	37.5	37.2	12.7	12.7	74.7	25.4	0.004	0	630969	6E + 05	2463	4548	371	69013	591	32	3	1
13	*Aphis nasturtii*	CP034888.1	37.7	37.4	12.4	12.4	75.1	24.8	0.003	0.001	630331	6E + 05	2379	4548	372	64522	584	31	3	1
14	*Aphis gossypii*	CP042426.1	37.4	37.2	12.7	12.7	74.6	25.4	0.003	0.002	628324	6E + 05	2464	4550	372	63466	583	32	3	1
15	*Aphis glycines*	CP009253.1	37.3	37.1	12.8	12.8	74.4	25.6	0.003	−0.001	628164	6E + 05	2458	4550	372	65088	581	32	3	1
16	*Pentalonia nigronervosa*	CP061275.1	37	36.8	12.9	13.2	73.8	26.1	0.002	0.01	617483	5E + 05	2470	4558	363	116502	522	32	3	1
17	*Hyadaphis tataricae*	CP034873.1	36.7	36.3	13.5	13.5	73	27	0.006	0.002	633867	6E + 05	2466	4553	370	67308	580	32	3	1
18	*Diuraphis noxia*	CP013259.1	37.5	37.1	12.7	12.8	74.6	25.5	0.005	0.006	636266	6E + 05	2461	4550	366	69455	586	32	3	1
19	*Lipaphis pseudobrassicae*	CP034870.1	37.5	37.1	12.4	12.5	74.6	24.9	0.004	0.004	641221	5E + 05	2477	4552	371	92222	578	32	3	1
20	*Brevicoryne brassicae*	CP034882.1	37.7	37.3	12.5	12.6	75	25.1	0.006	0.003	645850	6E + 05	2469	4549	369	68457	596	32	3	1
21	*Brachycaudus cardui*	CP034879.1	37.6	37.1	12.6	12.7	74.7	25.3	0.007	0.004	643931	6E + 05	2478	4551	363	65474	591	32	3	1
22	*Myzus persicae*	CP002699.1	37.5	37.1	12.7	12.8	74.6	25.5	0.006	0.005	643502	6E + 05	2479	4555	363	63744	586	32	3	1
23	*Artemisaphis artemisicola*	CP034900.1	38	37.5	12.3	12.3	75.5	24.6	0.006	0.001	633406	6E + 05	2478	4550	359	70038	577	32	3	1
24	*Hyperomyzus lactucae*	CP034876.1	37.2	36.7	13	13.1	73.9	26.1	0.006	0.004	641856	6E + 05	2487	4553	363	66886	591	32	3	1
25	*Macrosiphoniella sanborni*	CP034864.1	38.1	37.6	12	12.2	75.7	24.2	0.006	0.007	621931	5E + 05	2464	4571	366	82291	544	32	3	1
26	*Uroleucon sonchi*	CP047588.1	38.1	37.6	12.2	12.2	75.7	24.4	0.006	−0.001	614349	5E + 05	2464	4563	364	80215	539	32	3	1
27	*Uroleucon ambrosiae*	CP002648.1	38.2	37.7	12	12.1	75.9	24.1	0.007	0.002	615380	5E + 05	2464	4563	368	75953	541	32	3	1
28	*Sitobion avenae*	CP034855.1	37.2	36.8	12.9	13	74	25.9	0.006	0.005	636177	6E + 05	2479	4552	368	74738	571	32	3	1
29	*Sitobion miscanthi*	CP084934.1	37.2	36.9	12.9	13.1	74.1	26	0.004	0.008	671355	6E + 05	2479	4549	736	81297	601	32	3	2
30	*Acyrthosiphon kondoi*	CP002645.1	37.4	36.9	12.8	12.9	74.3	25.7	0.006	0.006	641794	6E + 05	2469	4559	363	73265	581	32	3	1
31	*Acyrthosiphon pisum*	BA000003.2	37.1	36.6	13.1	13.2	73.7	26.3	0.006	0.006	640681	6E + 05	2477	4549	366	71806	582	32	3	1
32	*Acyrthosiphon lactucae*	CP034891.1	38	37.5	12.1	12.3	75.5	24.4	0.007	0.008	642335	6E + 05	2478	4548	362	80073	573	32	3	1
33	*Microlophium carnosum*	CP048747.1	37.5	37	12.7	12.8	74.5	25.5	0.006	0.005	642296	5E + 05	2476	4551	364	89298	569	32	3	1
34	*Macrosiphum gaurae*	CP034867.1	37.3	36.9	12.8	13	74.2	25.8	0.005	0.008	643561	6E + 05	2474	4550	366	72885	581	32	3	1
35	*Macrosiphum euphorbiae*	CP033006.1	37.5	37	12.7	12.9	74.5	25.6	0.007	0.007	645334	6E + 05	2473	4550	364	76716	577	32	3	1
36	*Formosaphis micheliae* 27	*CP135045	38.3	38.2	11.6	11.9	76.5	23.5	0.001	0.012	554708	4E + 05	2536	4586	362	106692	457	33	3	1
37	*Formosaphis micheliae* 28	*CP135047	38.3	38.2	11.6	11.9	76.5	23.5	0.001	0.011	554353	4E + 05	2631	4587	362	109421	453	34	3	1
38	*Baizongia pistaciae*	AE016826.1	37.1	37.5	12.7	12.7	74.6	25.4	−0.006	0.001	615980	5E + 05	2474	4574	364	104082	521	32	3	1
39	*Melaphis rhois*	CP033004.1	36.9	37.1	12.8	12.8	74	25.6	−0.003	0.002	616452	5E + 05	2455	4587	362	103857	542	32	3	1
40	*Schlechtendalia chinensis*	CP011299.1	37	37.2	12.9	12.9	74.2	25.8	−0.002	0.003	607835	5E + 05	2462	4573	355	89734	547	32	3	1
41	*Mindarus japonicus* 20	*CP135030	38.4	38.3	11.6	11.7	76.7	23.3	0.002	0.002	541202	5E + 05	2618	4569	363	69261	476	34	3	1
42	*Mindarus keteleerifoliae* 18	*CP135024	38.1	37.9	12	11.9	76	23.9	0.003	−0.002	542558	5E + 05	2541	4572	366	74558	477	33	3	1
43	*Mindarus keteleerifoliae* 19	*CP135027	38.1	37.9	12	11.9	76	23.9	0.003	−0.002	542511	5E + 05	2610	4572	366	74688	476	34	3	1
44	*Taiwanaphis decaspermi* 29	*CP135049	39.7	39.1	10.6	10.6	78.8	21.2	0.008	0.001	453895	4E + 05	2389	4611	363	46053	411	31	3	1
45	*Neophyllaphis podocarpi* 25	*CP135039	38.7	39	11.2	11.2	77.7	22.4	−0.004	−0.001	559476	5E + 05	2387	4679	362	58020	519	31	3	1
46	*Neophyllaphis varicolor* 26	*CP135042	38.7	39	11.2	11.1	77.7	22.3	−0.004	−0.003	559524	5E + 05	2390	4670	362	59691	518	31	3	1
47	*Anoecia oenotherae*	CP033012.1	39.3	38	11.8	11	77.3	22.8	0.016	−0.036	548691	4E + 05	2637	4568	368	114458	444	34	3	1
48	*Thelaxes californica*	CP034852.1	39.1	38.3	11.4	11.2	77.4	22.6	0.011	−0.009	522699	4E + 05	2385	4698	383	76042	453	31	3	1
49	*Kurisakia onigurumii* 23	*CP135033	39.9	38.9	10.6	10.6	78.8	21.2	0.012	0	509028	4E + 05	2467	4342	382	62346	448	32	3	1
50	*Kurisakia onigurumii* 24	*CP135036	39.9	38.9	10.6	10.6	78.8	21.2	0.012	0	509341	4E + 05	2467	4669	383	61962	445	32	3	1
51	*Nipponaphis monzeni*	AP019379.1	39.2	38.5	11.2	11.2	77.7	22.4	0.009	−0.001	587781	4E + 05	2394	4599	355	145391	445	31	3	1
52	*Ceratoglyphina bambusae* 01	*CP134982	40.7	39.4	9.8	10.1	80.1	19.9	0.017	0.013	424619	4E + 05	2250	4557	356	45627	388	29	3	1
53	*Chaitoregma tattakana* 04	*CP134991	39.4	38.2	11.1	11.3	77.6	22.4	0.016	0.009	419464	4E + 05	2327	4636	364	41943	383	30	3	1
54	*Astegopteryx bambusae* 03	*CP134988	40.8	39.3	9.8	10.1	80.1	19.9	0.019	0.014	411922	4E + 05	2325	4636	366	39456	388	30	3	1
55	*Astegopteryx bambusae* 02	*CP134985	40.8	39.3	9.8	10.1	80.1	19.9	0.019	0.014	411372	4E + 05	2325	4633	366	38216	384	30	3	1
56	*Ceratovacuna japonica*	AP026065.1	40.9	39.1	9.9	10.1	80	20	0.022	0.012	414725	4E + 05	2323	4634	356	41805	379	30	3	1
57	*Ceratovacuna keduensis* 05	*CP134994	41.7	39.9	9.1	9.3	81.6	18.4	0.021	0.015	413541	4E + 05	2327	4637	360	39326	382	30	3	1
58	*Pseudoregma panicola* 07	*CP135000	41.3	39.6	9.4	9.7	80.9	19.1	0.021	0.014	412898	4E + 05	2324	4629	359	37591	383	30	3	1
59	*Pseudoregma panicola* 06	*CP134997	41.3	39.6	9.4	9.7	80.9	19.1	0.021	0.014	412562	4E + 05	2324	4629	359	37876	382	30	3	1
60	*Stegophylla sp*.	Manzano-Marín *et al*., 2023	38.4	38.6	11.5	11.5	77	23	−0.003	0.001	412953	3E + 05	2473	4565	369	68010	352	32	3	1
61	*Phyllaphis fagi*	Manzano-Marín *et al*., 2023	38.6	38.7	11.3	11.4	77.3	22.7	−0.001	0.002	431406	4E + 05	2548	4555	359	63425	370	33	3	1
62	*Shivaphis celti* 15	*CP134977	37.5	38.2	12.2	12.2	75.7	24.4	−0.009	0	422485	4E + 05	2391	4563	359	36404	388	31	3	1
63	*Therioaphis trifolii*	CP032996.1	39.8	40	10.1	10.1	79.8	20.2	−0.003	0.001	419293	4E + 05	2489	4551	364	33760	390	32	3	1
64	*Sarucallis kahawaluokalani*	CP032999.1	37.4	37.8	12.4	12.4	75.2	24.8	−0.004	0.002	428356	4E + 05	2389	4560	368	44242	390	31	3	1
65	*Takecallis taiwana* 16	*CP134978	37	37.3	12.8	12.9	74.3	25.7	−0.004	0.003	433453	4E + 05	2384	4554	364	43036	396	31	3	1
66	*Takecallis taiwana* 17	*CP134979	37	37.3	12.8	12.9	74.3	25.7	−0.004	0.003	432979	4E + 05	2384	4554	364	42601	395	31	3	1
67	*Greenidea ficicola* 11	*CP135012	40.7	40.7	9.3	9.3	81.4	18.6	0	0.004	395344	4E + 05	2234	4566	363	29894	368	29	3	1
68	*Mollitrichosiphum nigrofasciatum* 12	*CP135015	39	39.3	10.9	10.8	78.3	21.7	−0.005	−0.001	418955	4E + 05	2364	4557	335	50493	374	31	3	1
69	*Drepanosiphum platanoidis*	Manzano-Marín *et al*., 2023	41	40.6	9.2	9.2	81.6	18.4	0.004	0	448962	4E + 05	2398	4638	384	67208	379	31	3	1
70	*Periphyllus koelreuteriae* 21	*CP134980	40.7	41	9.2	9.1	81.7	18.3	−0.004	−0.003	452078	4E + 05	2324	4572	394	54044	408	30	3	1
71	*Periphyllus koelreuteriae* 22	*CP134981	40.6	41	9.2	9.2	81.6	18.4	−0.005	−0.004	451592	4E + 05	2325	4574	397	56471	406	30	3	1
72	*Sipha maydis*	CP097205.1	38.8	39.3	10.9	11	78.1	21.9	−0.007	0.003	462390	4E + 05	2401	4583	376	60308	411	31	3	1
73	*Periphyllus lyropictus*	CP097457.1	40.7	40.4	9.5	9.4	81.1	18.9	0.004	−0.009	456890	4E + 05	2314	4565	377	60459	411	30	3	1
74	*Nippolachnus piri* 09	*CP135006	39.4	39.6	10.5	10.6	79	21.1	−0.003	0.008	417640	4E + 05	2393	4570	384	47125	377	31	3	1
75	*Nippolachnus piri* 10	*CP135009	39.4	39.6	10.5	10.6	79	21.1	−0.003	0.008	417660	4E + 05	2393	4570	384	47145	377	31	3	1
76	*Tuberolachnus salignus*	LN890285.1	39.1	39.3	10.8	10.8	78.4	21.6	−0.003	−0.002	421426	4E + 05	2408	4578	388	47017	377	31	3	1
77	*Tuberolachnus salignus* 08	*CP135003	39.1	39.3	10.8	10.8	78.4	21.6	−0.003	−0.002	421666	4E + 05	2408	4578	388	47263	382	31	3	1
78	*Cinara confinis*	LT667503.1	37.9	38.1	11.9	12	76	23.9	−0.002	0.004	443747	4E + 05	2386	4573	387	68133	384	31	3	1
79	*Cinara tujafilina*	CP001817.1	38.4	38.5	11.5	11.5	76.9	23	−0.002	0.001	444925	4E + 05	2385	4575	384	78487	394	31	3	1
80	*Cinara cedri*	CP000263.1	40.1	39.8	10	10.1	79.9	20.1	0.003	0.004	416380	4E + 05	2390	4571	366	53361	369	31	3	1
81	*Cinara strobi*	LR025085.1	38.1	38	11.9	12	76.1	23.9	0.001	0.001	440140	4E + 05	2400	4580	374	67761	372	31	3	1
82	*Cinara piceae*	LR217739.1	39.2	38.9	11	10.9	78.1	21.9	0.004	−0.004	435017	4E + 05	2392	4595	375	65225	372	31	3	1
83	*Cinara curtihirsuta*	LR217700.1	39.5	39.3	10.6	10.6	78.8	21.2	0.002	0	433229	4E + 05	2390	4591	377	59409	375	31	3	1
84	*Cinara curvipes*	LR217710.1	39.5	39.3	10.6	10.6	78.8	21.2	0.002	0.001	433837	4E + 05	2394	4587	374	59504	376	31	3	1
85	*Cinara cuneomaculata*	LR217695.1	38.3	38.1	11.8	11.8	76.4	23.6	0.003	−0.001	430960	4E + 05	2476	4581	375	66837	368	32	3	1
86	*Cinara kochiana kochiana*	LR217707.1	38.5	38.2	11.6	11.7	76.7	23.3	0.004	0.003	433740	4E + 05	2386	4585	373	62421	374	31	3	1
87	*Cinara laricifoliae*	LR217717.1	39	38.7	11.1	11.2	77.7	22.3	0.003	0.002	436713	4E + 05	2394	4586	375	65617	374	31	3	1
88	*Cinara pseudotaxifoliae*	LT635893.1	38.1	37.8	12.1	12	75.9	24.1	0.004	−0.003	446627	4E + 05	2395	4578	369	70648	378	31	3	1
89	*Cinara cf splendens*	LR217692.1	38.2	37.8	12	11.9	76	23.9	0.005	−0.005	444797	4E + 05	2393	4575	370	70934	378	31	3	1
90	*Cinara splendens*	LR217722.1	38.4	38	11.9	11.7	76.4	23.6	0.006	−0.007	445237	4E + 05	2389	4577	371	69992	377	31	3	1

Asterisk (*) indicates that the genome was newly sequenced in our study.

### Genome characteristics

**Fig. 1:**
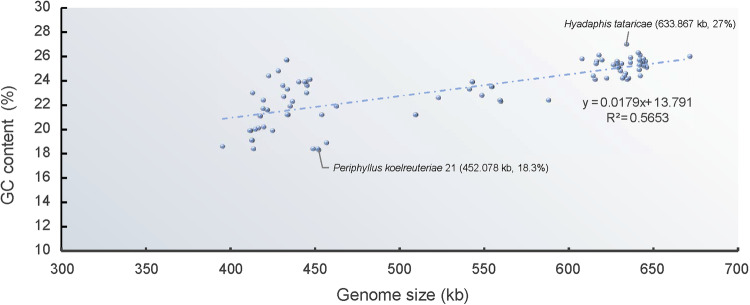
Relationship of genome size (kb) to GC content (%) in *Buchnera* genomes. *Buchnera* from *Hyadaphis tataricae* strain has the highest GC content (27%), *Buchnera* from *Periphyllus koelreuteriae* 21 has the lowest GC content (18.3%).

**Fig. 2:**
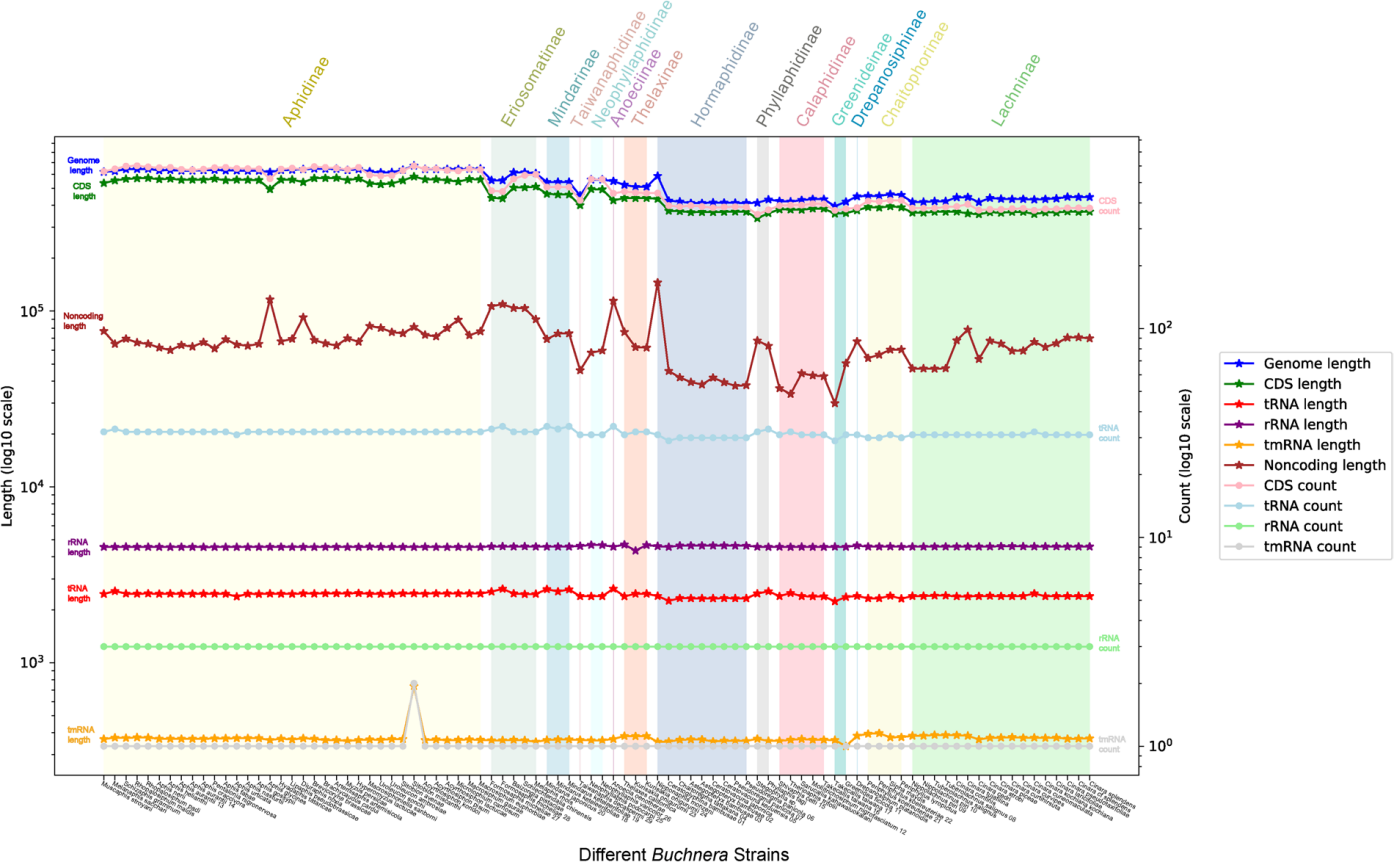
The length (indicated by asterisks) and number (indicated by circles) of various gene types in different *Buchnera* strains, as well as the length of the genome and non-coding regions. Different groups are represented by different colored lines. Various background colors represent *Buchnera* from different subfamilies of aphids, labeled with text in corresponding colors. The order of *Buchnera* strains from left to right corresponds to the order on the phylogenetic tree. CDS: coding sequence, mRNA: messenger RNA; tmRNA: transfer mRNA.

The PhyloSuite v1.2.1^36^ was used to extract the genomic feature information based on the well-annotated detailed files in GenBank format. Linear analysis was conducted to examine the relationship between genome size and GC content of the 90 *Buchnera* genomes. The genome size of *Buchnera* is from 395,344 bp (*Buchnera* from *Greenidea ficicola 11*) to 671,355 bp (*Buchnera* from *Sitobion miscanthi*), the average size is 531,263 bp (Table 1). The genome sizes of *Buchnera* across the Aphididae are larger than 600 kb, which representing the largest genome size observed among all *Buchnera* strains (Table 1). In contrast, the *Buchnera* strains from Hormaphidinae aphids display the smallest genome size (except *Buchnera* from *Nipponaphis monzeni* with 587,781 bp), only about 410 kb. Extreme genome reduction has been also confirmed in our newly sequenced *Buchnera* genomes, that of Hormaphidinae, Greenideinae, Chaitophorinae, Calaphidinae and Lachninae. And the *Buchnera* from *Greenidea ficicola 11* (Greenideinae), which encodes only 363 proteins, is the smallest *Buchnera* genome by far (395,344 bp). The length and the number of different type genes in different *Buchnera* strains have been shown in Table 1. We can find that the number and length of tRNA, rRNA, tmRNA are relative stable in different *Buchnera* strains, but the genome length, CDS (coding sequences) length and count, and noncoding length were significantly different. The average percentage of noncoding regions of all *Buchnera* strains account for 12.5% of the total genome size. The GC content of all *Buchnera* is very low, from 18.3% (*Buchnera* from *Periphyllus koelreuteriae 21*, with genome size 452,078 bp) to 27% (*Buchnera* from *Hyadaphis tataricae*, with genome size 633,867 bp), and the average is 23.3% (Table 1 and Fig. 1). The number and size of different types of genes in the *Buchnera* genomes from all aphid subfamilies were extracted. Additionally, the size of non-coding regions was also extracted. The reduced genomes tend to have a smaller number of genes (Fig. 2). Although some *Buchnera* strains still have relatively large genome sizes (e.g., *Buchnera* from *Nipponaphis monzeni* and *Anoecia oenotherae*), their noncoding regions are larger (reaching 24.7% of the genome size) than others.

### The COG categories classification of all *Buchnera*

**Fig. 3:**
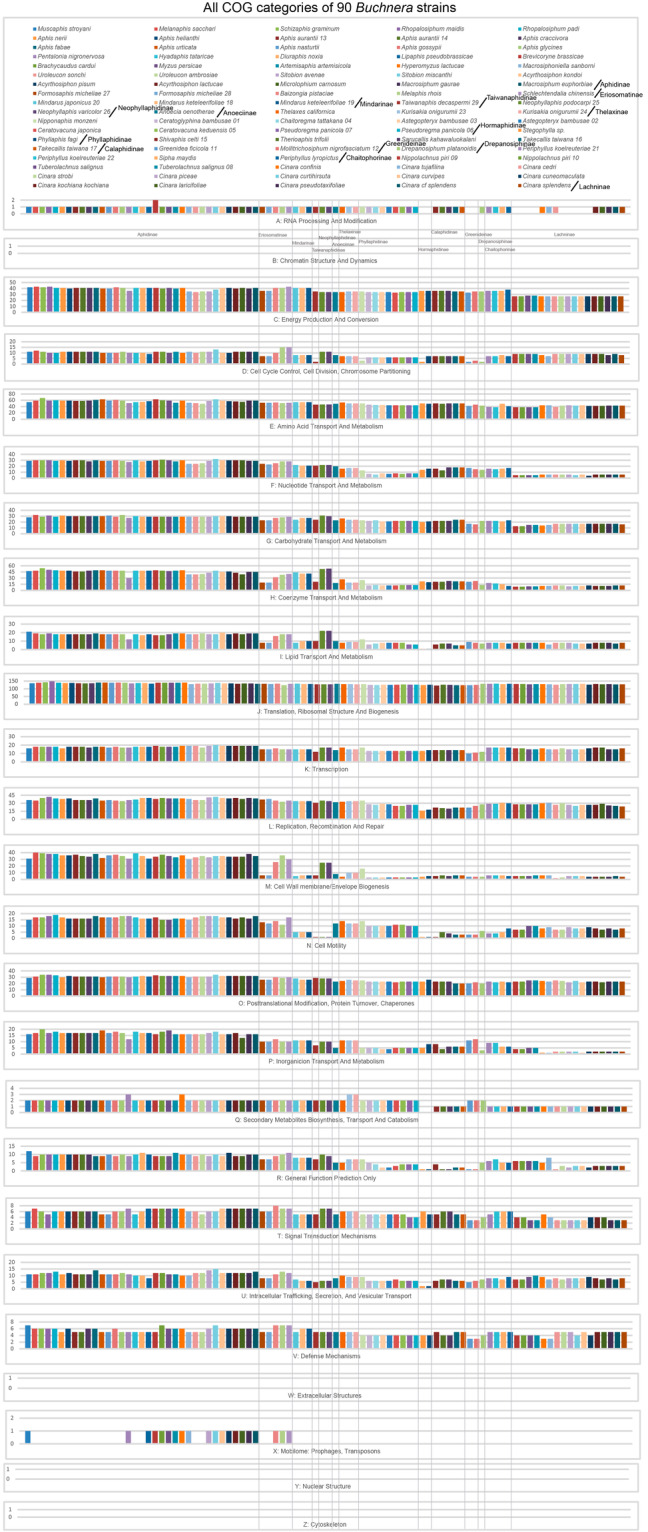
The gene clustering patterns of all *Buchnera* strains within different COG functional categories. Different colors represent different *Buchnera* strains within each category, with different subfamilies labeled in bold black text.

The protein coding genes presenting in all *Buchnera* strains were classified into different functional categories based on previous study^25^. The two datasets of clusters of orthologous genes (COGs) of *Buchnera aphidicola* and *Escherichia coli* K-12 substr. MG1655 (https://www.ncbi.nlm.nih.gov/research/cog/) were downloaded for the COGs annotation of all *Buchnera* strains. All the genes were classified into different COG categories based on the two datasets. Functional categories of all *Buchnera* genomes was presented in Supplemental Table S2. All protein coding genes was conducted to identify orthologous gene clusters across all *Buchnera* strains. The clustering of 26 functional categories showed the distinct patterns of orthologous gene reduction during the evolution of *Buchnera* (Fig. 3 and Supplemental Fig. S1). But there was no gene grouped into the follow functional categories: B (Chromatin Structure and Dynamics), W (Extracellular Structures), Y (Nuclear Structure), and Z (Cytoskeleton). Nearly all COG categories have an ongoing reduction of orthologous gene clusters from *Buchnera* strains with big genome size to small genome size.

## Data Records

The newly assembled genomes of 29 *Buchnera* strains (with strain names ending in numbers) are available through figshare^37^ as well as the National Center for Biotechnology Information (NCBI)^38–66^. The 29 mitogenomes of their host aphids, along with the comprehensive dataset based on 90 *Buchnera* genomes, can also be accessed through figshare^37^.

## Technical Validation

### Sequencing data quality control

In sequenced raw reads, low-quality sequences are present. To ensure the quality of bioinformatics analyses, the raw reads undergo filtering to obtain clean reads for subsequent bioinformatics analysis. Initially, Trimmomatic software is employed to filter raw tags and obtain high-quality sequencing data (clean tags). The clean reads data is obtained after the quality control of sequenced data.

### Validation of genome assembly

We utilized NOVOPlasty v3.5 and MEGAHIT v1.1.1 for sequence assembly. The sequences from different software were aligned with other complete sequences of *Buchnera*. If the similarity is lower than 50%, these sequences will be excluded in the future genome assembly. The analysis suggested completeness ranging from 92.96% to 99.99%, with an average of 98.36%. The relatively lower completeness of some smaller genomes is due to the genome reduction in *Buchnera*, a widely recognized phenomenon.

### Dataset quality control

The genome available in GenBank may not be complete or contain too many degenerate bases. Therefore, data cleaning is essential for construction of the comprehensive dataset and subsequent analyses. Initially, we conducted genome integrity assessments on all genomes downloaded from the GenBank database by CheckM2 v1.0.1^67^. The genome with low completeness were removed, such as the *Buchnera* genome (CP033006) from *Hormaphis cornu* (completeness: 64.04%, coding density: 50%, genome size: 643,250 bp). The base composition was analyzed using BioEdit v7.0.5.3^68^. Genomes with degenerate base content exceeding 1% of the total genome length were excluded. This step was crucial, as it may influence the subsequent analyses about genome length and the prediction of functional genes.

### Supplementary information

Supplemental Fig. S1

Supplementary Table S1

Supplementary Table S2

## Data Availability

In the absence of explicitly provided parameters, default configurations were applied to all softwares and tools throughout this study. No specialized code or script was employed in the research methodology.
