# Supplementary material for: Twenty-nine newly sequenced genomes and a comprehensive genome dataset for the insect endosymbiont Buchnera
Source: Sci Data. 2024 Jun 22;11:673. doi: 10.1038/s41597-024-03537-0 (PMC11193766; doi:10.1038/s41597-024-03537-0)
Supplement: Supplementary file 1 — Supplemental Fig. S1 [file 41597_2024_3537_MOESM1_ESM.pdf]

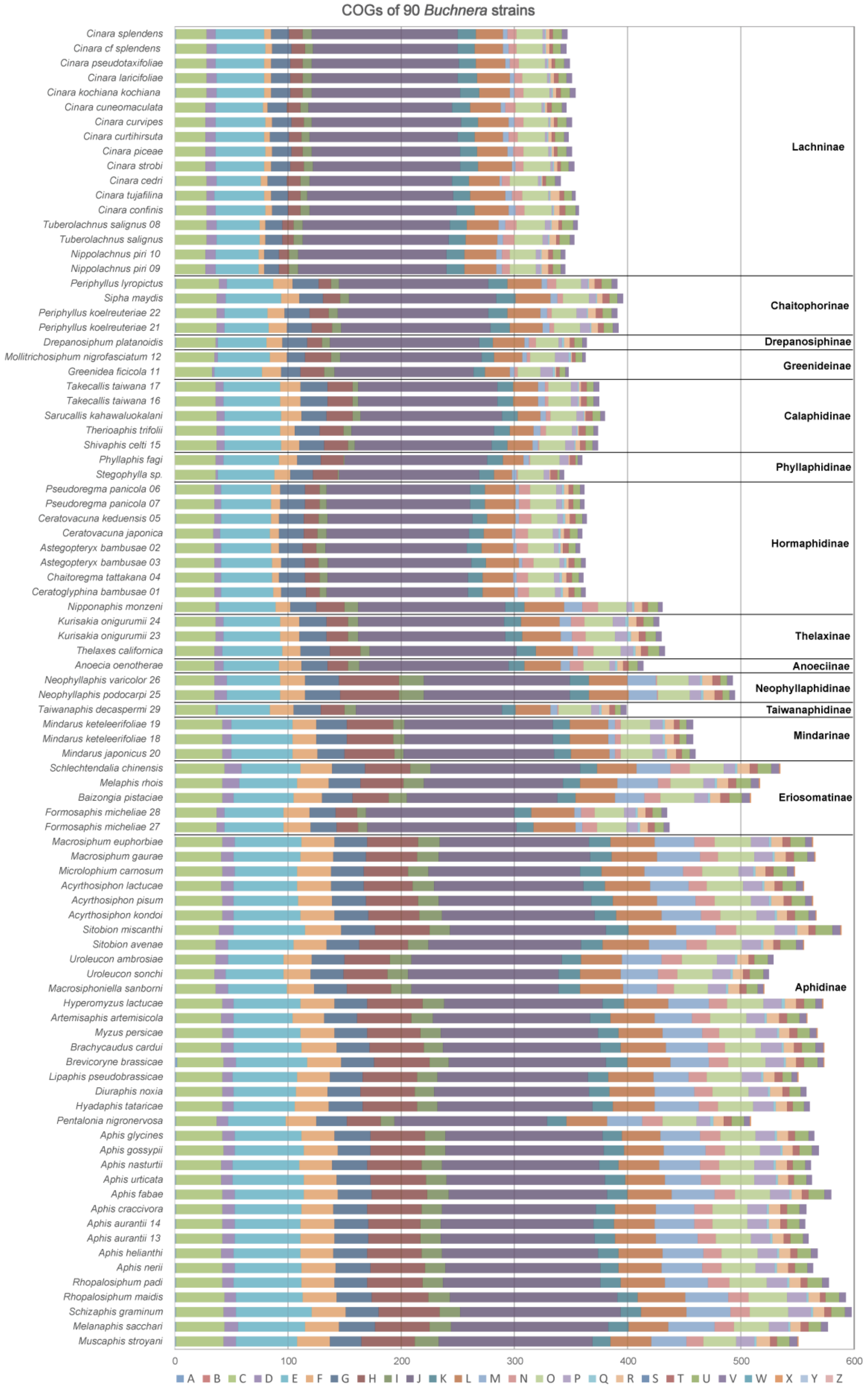

Supplemental Fig. S1 COG (Cluster of Orthologous Groups) analysis of protein-coding

genes for all *Buchnera* strains. The stacked bar chart, from left to right, represents categories A to Z, with different colors indicating various functional categories. The detailed annotations for categories A to Z are provided in figure 3. The order of *Buchnera* strains from bottom to top corresponds to the order from left to right in the figure 3, with different subfamilies labeled in bold black lettering.
